# Co-Designing Health Service Evaluation Tools That Foreground First Nation Worldviews for Better Mental Health and Wellbeing Outcomes

**DOI:** 10.3390/ijerph18168555

**Published:** 2021-08-13

**Authors:** Michael Wright, Aunty Doris Getta, Aunty Oriel Green, Uncle Charles Kickett, Aunty Helen Kickett, Aunty Irene McNamara, Uncle Albert McNamara, Aunty Moya Newman, Aunty Charmaine Pell, Aunty Millie Penny, Uncle Peter Wilkes, Aunty Sandra Wilkes, Tiana Culbong, Kathrine Taylor, Alex Brown, Pat Dudgeon, Glenn Pearson, Steve Allsop, Ashleigh Lin, Geoff Smith, Brad Farrant, Leanne Mirabella, Margaret O’Connell

**Affiliations:** 1 School of Allied Health, Curtin University, Bentley, Perth 6102, Australia; m.wright@curtin.edu.au (M.W.); tiana.culbong@curtin.edu.au (T.C.); 2 Elders Co-Researcher Group, Perth 6000, Australia; dot.getta@outlook.com (A.D.G.); oriel.green@outlook.com (A.O.G.); charlie.kickett@outlook.com (U.C.K.); helen.kickett@outlook.com (A.H.K.); evelynmarch4@gmail.com (A.I.M.); albertmcnamara109@gmail.com (U.A.M.); newmanmoya61@gmail.com (A.M.N.); pellcharmaine@gmail.com (A.C.P.); millipenny@outlook.com (A.M.P.); peterwilkes09@gmail.com (U.P.W.); sandrawilkes332@gmail.com (A.S.W.); 3 Woodside (Australia), Perth 6000, Australia; kathrine.taylor@woodside.com.au; 4 South Australian Health and Medical Research Institute, University of South Australia, Adelaide 5001, Australia; alex.brown@sahmri.org.au; 5 Poche Centre for Indigenous Health, University of Western Australia, Crawley 6009, Australia; pat.dudgeon@uwa.edu.au; 6 Telethon Kids Institute, University of Western Australia, Nedlands 6009, Australia; glenn.pearson@telethonkids.org.au (G.P.); ashleigh.lin@telethonkids.org.au (A.L.); brad.farrant@telethonkids.org.au (B.F.); 7 National Drug Research Institute, Curtin University, Bentley, Perth 6102, Australia; s.allsop@curtin.edu.au; 8 Medical School, University of Western Australia, Crawley 6009, Australia; geoff.smith@uwa.edu.au; 9 Independent Consultant, Perth 6000, Australia; leannemirabella@westnet.com.au

**Keywords:** first nations, co-design, Indigenous research methodologies, service evaluation, participatory action research, relationships, engagement, worldviews

## Abstract

It is critical that health service evaluation frameworks include Aboriginal people and their cultural worldviews from design to implementation. During a large participatory action research study, Elders, service leaders and Aboriginal and non-Aboriginal researchers co-designed evaluation tools to test the efficacy of a previously co-designed engagement framework. Through a series of co-design workshops, tools were built using innovative collaborative processes that foregrounded Aboriginal worldviews. The workshops resulted in the development of a three-way survey that records the service experiences related to cultural safety from the perspective of Aboriginal clients, their carer/s, and the service staff with whom they work. The surveys centralise the role of relationships in client-service interactions, which strongly reflect their design from an Aboriginal worldview. This paper provides new insights into the reciprocal benefits of engaging community Elders and service leaders to work together to develop new and more meaningful ways of servicing Aboriginal families. Foregrounding relationships in service evaluations reinstates the value of human connection and people-centred engagement in service delivery which are central to rebuilding historically fractured relationships between mainstream services and Aboriginal communities. This benefits not only Aboriginal communities, but also other marginalised populations expanding the remit of mainstream services to be accessed by many.

## 1. Introduction

The importance of improving health service delivery to more effectively reflect and respond to Aboriginal and Torres Strait Islander (Aboriginal and Torres Strait Islander peoples is the inclusive term that refers to the First Nations Peoples of the nation state now known as Australia. This paper describes a project located in the metropolitan region of the south west of Western Australia where the term ‘Aboriginal’ is often preferred. Throughout this paper, the term ‘Aboriginal’ is respectfully used.) Communities through culturally secure care is well recognised [1,2,3,4]. Increasingly, on a global scale, it is also acknowledged that involving communities in co-designing healthcare strategies via equal partnership, participation and decision-making will have enormous benefits, from developing programs that are more culturally responsive and secure to building better relationships between researchers, services and communities [5,6,7,8,9,10,11,12].

In Perth, Western Australia (WA), a large Participatory Action Research project, led and guided by Aboriginal Elders, has been undertaken to improve the way mainstream mental health and drug and alcohol services are accessed by, and are responding to, Aboriginal people. The *Looking Forward Moving Forward* project takes place in the metropolitan area of Perth, Western Australia. There are 10 partner organisations involved in the five-year study. Each partner organisation has made a financial commitment to the project. The WA Mental Health Commission is responsible for the purchase of state-wide mental health services in the non-government sector and is a key partner on the project.

This article describes a series of workshops co-designed by Aboriginal Elders and service staff and non-Aboriginal service personnel, including executive and practitioner staff, and Aboriginal and non-Aboriginal researchers. It details the principles that underpin the co-design process, framed by decolonising research methodologies that may be of benefit to other researchers working alongside First Nation communities and other vulnerable population groups. The objective was to co-design impact measures that would capture organizational change due to the Elders’ interaction with service leaders over a lengthy period of time, with many of the partner organization’s personnel having developed relationships with the Elders for at least four years. Three workshops were held during which participants collaborated to construct the study’s assessment procedure and the survey tool used to measure the impact of organizational transformation on Aboriginal clients’ health and well-being outcomes. The survey tool will be used by the partner organisations after the project has been completed and will, we believe, have wider applicability for other service providers working with Aboriginal people, both within WA and Australia. 

### Context

**Figure 1:**
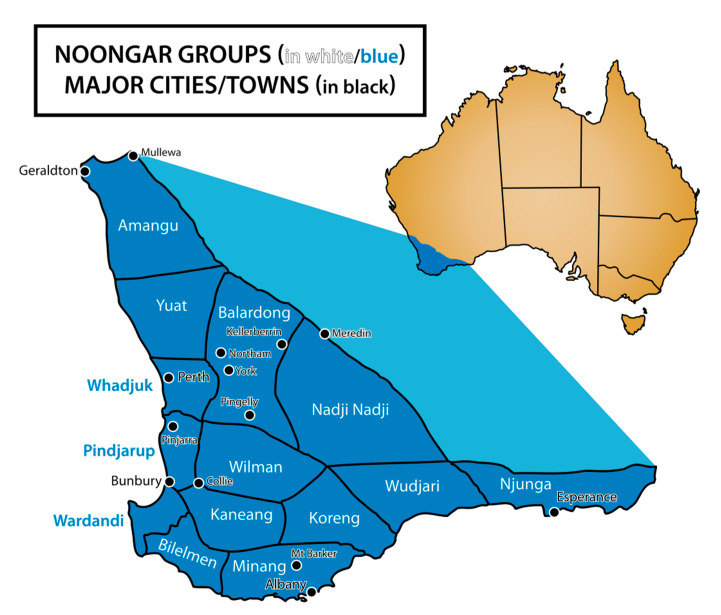
Nyoongar groups that make up the Nyoongar Nation in the lower southwest of Western Australia. Image by Brooke Ottley. Reproduced under the Creative Commons Attribution—Share Alike 3.0 Unported License, 2011. Image source: https://en.wikipedia.org/wiki/File:Noongar_regions_map.svg (accessed on 29 June 2021).

The project is conducted with Nyoongar people, the Aboriginal people of the South West of WA, on *Wadjuk Nyoongar Boodja* (Country). *Wadjuk* is one of 14 *Nyoongar* language groups of the region. *Nyoongar Boodja* covers approximately 200,000 square kilometres of the South West (see Figure 1). 

The Australian Bureau of Statistics (ABS) estimated that in 2016, 40,482 Aboriginal and Torres Strait Islander people lived in Perth and the South-West region, constituting 40.3% of the total Western Australian Aboriginal and Torres Strait Islander population [13]. The South West Aboriginal Land and Sea Council estimates the Nyoongar population to be approximately 30,000 [14]. 

Twenty-two Elders living across the Perth metropolitan area agreed to participate as co-researchers. Of these, fifteen worked directly with one or more of the partner organisations. There are ten partner organisations including the WA Mental Health Commission, three peak agencies, one large hospital, four mainstream non-government mental health service providers and two alcohol and other drug support services. Chief investigators include Aboriginal and non-Aboriginal scholars with diverse health research backgrounds including health economics, policy and strategy, Aboriginal research methodologies and community engagement, suicide and self-harm prevention, clinical and psychosocial mental health and wellbeing, and two medical doctors. 

## 2. Background Literature

### Aboriginal Lived Experiences of Mental Health and Wellbeing

Aboriginal and Torres Strait Islander people have a different worldview about mental health and wellbeing as compared to non-Indigenous people. Their experiences are predicated on the impact of colonisation and intrinsically linked to kin, culture and Country [15,16,17,18,19,20]. However, mainstream mental health services are predominantly structured in western ways that do not easily respond to the lived experiences of Aboriginal and Torres Strait Islander people seeking support for mental health concerns [21,22]. Culturally safe, mental health care needs to include more holistic and inter-connected understandings about self-determination, empowerment, identity, family and community, spirituality, Country, speaking language, cultural practices, and physical health [23,24,25,26,27]. Relationships are central to Aboriginal people, and it is the wholeness of these relationships rather than the abstract nature of each component that is key to wellbeing and lived experience.

Mental health and wellbeing are of paramount concern for Nyoongar people [28]. However, the cultural inappropriateness of services, experienced by Aboriginal clients as the ongoing impacts of colonisation, means that they are not accessed proportionately to their needs and the community therefore remains dangerously underserviced [28]. The *Looking Forward Moving Forward* project aims to improve mainstream service delivery by changing organisational practice through the direct engagement with Aboriginal Elders and sustaining these changes through ongoing community partnerships with service organisations. A key objective of the study is to co-design a service evaluation that provides an evidence base for understanding the ways in which Elders and community members can drive organisational change as a result of their direct engagement with services leaders and staff [27,29,30,31]. 

Central to improving service delivery is evaluating how well services are responding to Aboriginal clients’ needs and the way services might incorporate Aboriginal and Torres Strait Islander meanings of health and wellbeing [32,33,34,35]. While evaluations can support services to redesign and improve their programs, if the evaluation is not effectively assessing their activities based on the needs of the *lived experience* of Aboriginal clients, the findings are likely to be inappropriate and incompatible. This can result in ineffective service-based changes that will not help build trust between communities and services, ultimately perpetuating the ongoing cycle of inequality and poor health outcomes. Unfortunately, most evaluation instruments are unsuitable for, or irrelevant to, their local context, with minimal scope to acknowledge and work from the lived experience and cultural worldview of Aboriginal clients, limiting opportunities for Aboriginal collaboration and contribution [36].

Aboriginal evaluation frameworks are an emerging field with some important projects and contributions. For example, the *Ngaa-bi-nya* Framework is a practical evaluation guide that was designed from an Aboriginal standpoint informed by the holistic concept of Aboriginal health and used a mixture of quantitative and qualitative methods [37]. Co-designing evaluations can strengthen, improve and provide a sense of ownership for community members [38,39,40,41,42]. Foregrounding Aboriginal ways of working ensures evaluation instruments are relevant, credible and importantly, effective and meaningful in documenting outcomes [42,43]. Here, the vision is not activating evaluation as a means to an end, but rather achieving long-term sustainable benefits through deepening relationships and community inclusion, where more strengths-based evaluation processes as well as outcomes align with community priorities and their preferred ways of working [28,33,41,42].

## 3. Decolonising Methodology: Engaging the Wisdom of Aboriginal Elders

Decolonising research demands that Aboriginal people sit at the centre of the design, delivery, interpretation and translation of research and evaluation endeavours [43,44,45,46]. It places Aboriginal knowledges, lands and cultural practices at its heart. In privileging Aboriginal ways of working, decolonising research entails more than the written word; it includes, for example, yarning, sharing stories, artwork, on Country activities, sharing cups of tea in kitchens, and convening large gatherings of Elders and family members in local community centres or parks. Research can be a powerful catalyst for participants but only if they remain in control of their stories and the way in which they choose to tell them as part of the research journey [47].

Key features of decolonising research methodology relevant to our study are co-design, Aboriginal governance and leadership and the privileging of Aboriginal worldviews. Co-design strategies promote collaborative leadership, trusting relationships and shared power [19,31,41,48,49]. As a decolonising approach, co-design facilitates the sharing of stories and directly hearing community voices about their lived experiences. These are, as Kendall and colleagues state, stories to be “recognized as precious belongings, not something to be dissected and reinterpreted out of context” ([47], p. 268). Thus, the stories and experiences shared by the Elder co-researchers were interwoven with connections to family, history, kin and Country [28]. 

In the case of this study, it has been through the Elders that the privileging of Aboriginal worldviews is foregrounded and sustained. Aboriginal people’s worldviews are experiential and intricately relational, with meaning made through reciprocity, kinship and relationships that are fully inclusive and highly contextual. As leaders, Elders are crucial in ensuring the cultural, social and emotional wellbeing of their community. Their cultural authority and status [28,29,43,50,51,52] mean they are key conduits for building relationships between Aboriginal communities and mainstream health services. In terms of Aboriginal governance, the study applies an Aboriginal-led research methodology grounded in participatory action research [10,11,12,28,29,41,52] and is held by the Elders through their cultural wisdom and guidance [51,52]. The Elder co-researcher group performs a number of leadership roles within the project. Firstly, they are central to the governance of the project. The chief investigator team and the project team have been guided by the Elders in the design and development of the research and evaluation. In addition, the project team have engaged an Elder-in-residence to enhance their cultural supervision and support. Secondly, they ensure the Aboriginal community is appropriately represented. Thirdly, the Elders hold the researchers and mainstream service partners to account by promoting community imperatives and facilitating shared spaces in which learning and understanding about Aboriginal culture and history can occur. In addition, the Elders share their experiences and play an active and meaningful role in their own, and their community’s, mental health care. The Elders not only promote the community’s priorities, but also help to establish a shared space for learning that brings together Aboriginal ways of working and western ways of undertaking research and evaluation. Furthermore, and finally, in working together, Elders and service organisations can be more proactive in translating the study findings into practice and policy across the state.

Subsequently, the co-design phase of the study wove together three defining relational elements—*place, people* and *positionality*. That is, the co-design workshops were held in places of cultural significance and each began with a Welcome to Country (A ritualised protocol whereby an Elder of the local Aboriginal language group with which the land that the particular meeting/event is being held on formally welcomes visitors to their traditional lands) performed by an Elder/s. The Welcome to Country was followed by an introductory presentation from the Aboriginal lead Investigator (MW) to ‘set the scene’ and intentions for the workshop. 

Each of the workshops were held in June (the Nyoongar season of *Makuru*), August (the season of *Djilba*) and October (the season of *Kambarang*) of 2019 and paid homage to the seasonal cycle important to Nyoongar people. (The Nyoongar cycle includes six seasons; Makuru, Djeran, Kambarang, Djilba, Birak and Bunuru. Each season is punctuated by the growth cycles of local plants and the movement of local fauna. The shift in weather patterns also determines the seasonal cycle. Nyoongar people would move across the Country prompted by the seasons and the food and materials each season provided them, intricately linked in relationship to flora and fauna around them across the landscape). An inclusive approach was adopted in the workshops with stories by the Elders being central to the discussions. Discussions in the workshops were held as ‘yarns’ or ‘storying’; an Aboriginal cultural way of communicating. Yarning, or storying privileges, Aboriginal voices and creates the necessary foundations for relationships to be built [53,54,55]. Roundtable group discussions, or yarns, were each facilitated by an Aboriginal co-investigator (AB, PD, GP) together with the Elders. Non-Aboriginal co-investigators (AL, SA, GS, BF, LM) and Aboriginal and non-Aboriginal research team members (MO, TC) supported the activities with their combined experience in research approaches, mental health and wellbeing, community engagement and evaluation.

### 3.1. Preparation for Co-Design Workshops

Prior to the co-design process in 2019, three thematic working groups, namely, *Cultural Security, Workforce* and *Governance,* were established and met bi-monthly during 2018. Elders and service leaders were the key collaborators for these working groups. The three groups were formed based on analysis of qualitative data collected in 2017 via semi-structured interviews with Elder co-researchers and service partner staff. Members from the three working groups identified the key aims and objectives that directed and guided the co-design workshops held in 2019. During the co-design workshops, these aims and objectives were reviewed as the “draft evidence statements” by which measures could be developed.

The co-design workshops were led by the investigator team who directed the service evaluation. The investigator team facilitated the co-design workshops between Elders, service leaders and service staff to design both the evaluation process and the evaluation tools that will be used to assess the cultural responsiveness and effectiveness of service delivery to Aboriginal clients. The overall evaluation will measure the impact of the engagement between Elders and the service leaders and the resulting organisational change on the service experiences of Aboriginal clients.

The three workshops were held in locations across the Perth metropolitan area to which the Elder co-researchers recounted cultural stories for the benefit and learning of non-Aboriginal co-researchers. Most of the same participants were present at each of the three workshops. The research team acted as secretariat for the workshops and incorporated participant feedback in between each of the workshops which in turn helped to shape the agenda for each.

### 3.2. Makuru: Workshop One

The first workshop revisited the themes of *Governance, Workforce* and *Cultural Security* formed during the 2018 working groups. This enabled participants to re-familiarise themselves with the intentions behind the themes and the corresponding strategies and actions developed from them (see Table 1). These strategies and actions were recast as “draft evidence statements” to be reviewed as potential measures by workshop participants. Two Elders, 13 service staff, 10 research investigators and seven research team members attended the workshop. In the context of the three key themes, the participant groups were tasked with developing outcome indicators aligning with each of the following questions: *(i) What would a service that is culturally safe for Aboriginal people look like? (ii) What should we measure to see change in a service? (iii) How do we measure these changes in a service?*

### 3.3. Djilba: Workshop Two

Eight Elders, 11 service staff, four investigators and seven research team members attended Workshop Two. The “draft evidence statements” reviewed by participants in Workshop One were ranked by participants in order of priority and their ability to be measurable (see Table 1). At this workshop, participants also agreed on the most appropriate format for collecting service data and client service experiences.

In between Workshop One and Workshop Two, Aboriginal research team members undertook a targeted review of pre-existing service experience and mental health and wellbeing measures and survey tools. Instruments validated by Aboriginal people were given priority [56,57,58]. Other instruments not been specifically validated for (nor by) Aboriginal people and used by services were reviewed and also considered [59,60]. The selected measures were then workshopped with the research team, examining features such as the holistic/multi-dimensional view of measures (for example, inclusive of spirit, culture, family, and sense of self) their strengths-based focus, their relevance and adaptability, and their ability to acknowledge and reflect the relationships between service staff and Aboriginal clients. 

### 3.4. Kambarang: Workshop Three

Workshop Three involved presenting the review of draft survey tools to the co-design participants for feedback. Samples of the reviewed tools were provided to participants and some participants role-played the use of these to establish a greater understanding of their purpose and outcomes. Twelve Elders, 16 service staff, four investigators and seven research team members attended the workshop. Service-level orientation and training strategies were also discussed.

### 3.5. Analysis and Consensus Building

The co-design was driven by consensus building and a voting process was used, underpinned by Aboriginal voices, punctuated by goodwill, respectful humour and sharing of food. Voting and consensus building had proven effective in an associated project [55]. Consensus building was considered an appropriate means to deriving agreement by acknowledging the diversity of voices present during the workshops, privileging Aboriginal voices. Consensus building is a highly engaged process that involves alignment of intentions, shared commitment and shared expertise that acknowledges both service and community skills and knowledge [61,62]. The collective wisdom and authority of the Elders attending these workshops provided a community-driven focus on developing consensus and agreement, whereby Elders in particular would share stories with historical references as well as anecdotal references to community lived experiences. Community and cultural values such as respect, relationship and trust underpinned this process. 

Participants were each given nine voting stickers and asked to register their vote next to the measure they felt demonstrated a change in organisational behaviour and practice and/or had a measurable client/community outcome as a result of change. It was agreed by the participants that for the voting process the Elders’ votes would be double weighted in recognition of their status and unique contribution to the process. As there were twice as many non-Aboriginal participants as Elders, voting scores were weighted one point per service staff vote and two points for each Elders’ vote, with researcher votes included in the tally of service staff scores. Total scores were counted according to weighted votes, with overall scores against each statement ranked from highest to lowest for both Elders as well as for service staff.

A focus group was held after the workshops with a smaller number of co-design participants and included one Elder co-researcher, two Aboriginal Health Workers, research team members, a data analyst with client survey experience, and the lead investigator (MW). The focus group was intended to verify the outcomes and priorities from the workshops. A final review of existing survey tools was presented at this focus group to ensure a full coverage of client experience instruments, as gaps had been identified after the third and final workshop. In addition, during the final workshop the Elders had stated that organisational support for the workers engaging directly with clients was important to capture as part of the evaluation. A strengths-based approach was preferred and the research team undertook further instrument review to ensure these aspects were included.

## 4. Results

### 4.1. Workshop Outcomes 

Outcomes from Workshop One were presented as evidence statements. It was agreed that evidence statements must have a measurable quality to them. As described earlier, these were grouped under the themes of governance, workforce and cultural security that were formed during the 2018 working groups (Table 1).

Evidence of positive impacts on clients derived from the workshop information presented in workshop two included cultural connectedness, experience of service, community engagement, worker competence and confidence, and organisational governance and leadership. These themes aligned with the review of measures and instruments conducted by the research team. Most of the evidence statements the Elders had voted for strongly reflected their priority for relationships. Voting results also showed consensus about the importance of the employment of, and support for, an Aboriginal workforce. The same applied to straightforward, plain language cultural security statements, such as, “do you feel safe?” and “is the service welcoming/approachable?”

Workshop Three involved presenting the draft survey instruments for feedback. Earlier iterations had weighted heavily on questions that referred to cultural connectedness of the client and the worker. However, feedback at Workshop Three highlighted that greater consideration should be given to service experiences rather than a perceived assessment of a client’s connection to culture. Concerns about the length of the survey were expressed, as was the suggestion to use plain and concise language. A series of follow up meetings addressed these concerns. The draft survey tool was piloted with staff at one of the partner services. Feedback was positive, with some suggested amendments which were applied. The resulting survey package was then made available for trialing across the six service partners. 

Adopting a decolonising approach in a co-design process that includes Aboriginal people is essential. We believe it is a liberating and emerging field, rich with potential for developing impactful evaluation approaches and outcomes that are more culturally appropriate and thus responsive to the lived experiences of Aboriginal people. Whilst the intersection of divergent worldviews created some challenges in a space that is still very much dominated by Eurocentric views and ways of working, our experiences shared during the workshops has been that decolonising research spaces using co-design approaches can happen if the focus is on being respectful and authentic. This quote from a participating Aboriginal researcher highlights the unique way of working that comes from bringing diverse stakeholders together in this way:

*“…a lot of people aren’t even having the thinking like you guys are doing here. Because of the Looking Forward I think there’s been this great step forward. Yeah. It’s been a step forward whereas other places in Australia it’s very spasmodic. So you might have a little fire here that’s burning and doing good things”.*

(Aboriginal researcher, Workshop One)

### 4.2. Themes and Priorities

It was evident in the workshops across all the tables/groups that good service practices, responsive governance and a competent workforce all needed to align for effective practice when working with Aboriginal families. Feedback from participants highlighted the importance of measures that are strength-based; more holistic in nature encompassing impacts on family, community and culture (rather than confined to mental health) and which avoided asking too many culturally-direct and thus potentially invasive questions. The impact of wellness and self-esteem in both clients and service providers on the client’s service experience was also identified, and a number of self-reporting wellbeing tools were examined [25,63,64]. 

Elders highlighted that the service experience extended beyond the interaction between the worker and the client, and thus developing tools that recognised the critical role of family and caregivers in the clients’ experience was deemed necessary [56]. For example, during one co-design workshop, one of the Elders described the family member or support person as a key advocate, where “humanness is the bottom line” and they help to “keep clinicians respectful”.

From here emerged strong support for a triangulated, three-way survey that would be administered to clients, service workers and family members/significant others, to give a more holistic view of the client’s service experience by capturing these multiple perspectives. The following quote from one non-Aboriginal workshop participant highlights the connectedness of Aboriginal workers as one example of the interrelatedness of community, culture and work practices.

*“…really recognising the contribution that Aboriginal staff have and are making in terms of maybe it doesn’t quite ‘fit’ into the standard western society [job description form]. But it’s actually much more important in a lot of ways than this other stuff. Especially if we start to talk about people who are holding important relationships and managing the engagement with the families and the communities and stuff that’s actually enabling you know what used to be called ‘hard to reach’ people to actually engage with a service, realising just how critical that is”.*

(non-Aboriginal workshop participant, 2019)

Elders and Aboriginal service staff articulated the importance of strengths-based rather than deficit focused measures that “should lift people up,” as one Aboriginal health worker described during one of the co-design workshops, bringing to light a range of social, cultural and emotional indicators that may have been affected by the service they received. The intention was to create an evaluation tool that would not become another therapeutic assessment tool, but an attempt to identify from the client’s perspective how they perceived the worker had understood and recognised culture as a protective factor and critical in the client’s recovery journey [24,65,66].

Perhaps the most challenging aspects to develop measures and evaluation approaches concerned organisational governance. Organisational governance entails the stewardship and resourcing of an organisation’s mission, strategies and culture. One workshop participant reflected on the divergent discussions related to the theme of governance and the realisations the roundtable discussions came to:

*“…there were a whole lot of specifics that were about how would we pursue different strategies. But I think the point that we really got to at the end of it of course was we need to step back and say ‘Well, what is the intent of this governance in the first place?”.*

(Non-Aboriginal workshop participant, Workshop One)

## 5. Discussion

There is a recognised need for evaluation frameworks to be more reflective of, and responsive to, Aboriginal worldviews to allow the cultural standpoint of Aboriginal people to directly contribute to the quality improvement of mainstream health service delivery [29,37,38,39,40,41,42]. The development of the service evaluation is an example of how co-design that foregrounds Aboriginal ways of working can be more responsive and relevant to local cultural needs. Three main learnings are apparent as a result of the co-design workshops undertaken.

### 5.1. Engaging Directly and Regularly with Elders as Co-Researchers

Firstly, for service leaders, in order to enhance these imperatives, engaging directly and regularly with Elders as co-researchers is essential as they are the key for transformation to occur. They imbue an authority equal to that of the service leaders and can speak to the priorities of the Aboriginal community that temper the priorities of the service providers. This sends a strong message to the community and enhances the service’s visibility. The co-design process has provided new insights into the reciprocal benefits of engaging the Elders as community leaders and the service leaders to work together, *burdiya* to *burdiya*, (the Nyoongar term for “boss to boss”) to develop new and more meaningful ways of servicing Aboriginal families seeking support for their mental health and wellbeing. The presence of the Elders illuminates the cultural imperatives and value of cultural ways of being, doing and knowing that impact mental health and wellbeing [36,42,67].

### 5.2. A Shared Understanding about Taking a Strengths-Based Approach

Secondly, for Aboriginal and non-Aboriginal service staff alike, developing a shared understanding that taking a strengths-based approach to experiences of mental health and wellbeing and acknowledging the central role of culture as a protective factor in Aboriginal people’s recovery journeys is critical [17,65,66]. Strengths-based approaches lead to positive impacts for the community, based on their direct engagement and input into the research itself [33,38,42]. Having Elders in the workplace provides an additional protective factor that supports Aboriginal workers as well as clients and serves to refocus support efforts on client, family and community assets and strengths underpinned by cultural origins of good health and wellbeing to attend to concerns about mental ill-health. 

### 5.3. Service Staff Seek to Be Culturally Responsive but Do Not Know How

Thirdly, the Elders learnt that service staff are deeply committed to providing respectful and culturally responsive care but that many did not know how to go about achieving this [33,34,35,36]. To this end, the Elders were consistent in their views to ensure the organisation provided ongoing support to Aboriginal and non-Aboriginal health workers and that any service experience or satisfaction measures should capture the importance of supporting staff as well as clients through effective governance strategies. By explicitly acknowledging the skills and experience of Aboriginal and non-Aboriginal staff, organisations are demonstrating their value and worth as people who feel comfortable, safe and supported to bring their “whole selves to work”.

### 5.4. Role of Family and Community in Understanding Collective Experiences of Wellbeing and Recovery

Finally, the design of the survey to capture multiple perspectives of a client’s service experience acknowledges the critical role of family and community in understanding and integrating collective experiences of wellbeing and recovery [15,16,50,51,52,55,56,57,58,64,65,66]. This exemplifies the role relationships play in people’s lived experience being shared, connected, and central to a person’s sense of belonging and wellness. Coupled with more strengths-based discourse, these connections enable Aboriginal communities to see improvement and progress amidst the ongoing concerns about their social and emotional wellbeing [15,16,17,18,19,21,22,23,24,25,26].

We found that evaluation approaches must reflect these characteristics in order to appropriately capture outcomes relevant to a service provider’s way of working with Aboriginal service users. The challenge remains in achieving a balance between measures tailored to reporting and accountability and those that respond to the priorities outlined by the Elders and the community. Evaluation approaches can only do this if they are co-designed with service users who are set to benefit most from the results. The Aboriginal investigators offered further provocations to workshop participants to ensure that the measures developed were not developed for “measurement sake”, as this quote illustrates:

*“…We’ve talked about the human qualities or human interactions and relationships or the importance of culture within an organisation embodies through Aboriginal position, it’s probably not something that is measurable right? So I just want us to be cautious so you know? We can come up with all of these 20,000 things to measure and we should be choosing the things that matter most”.*

(Aboriginal researcher, Workshop One)

We believe these co-design efforts present a unique contribution by demonstrating the potency of centralising relationships between Indigenous cultural leadership and mainstream leadership. Co-design does this by minimizing the tendency to polarize the expertise and experiences manifested in different worldview understandings, instead cultivating equal partnerships that can actualise truly co-designed outcomes [28]. It is these relationships—and the continual nurturing and prioritising of these—which exemplifies Aboriginal worldviews to provide a platform from which any effective work will develop. 

The intention in this paper is not to describe the survey tool in depth, but instead to capture the essential principles that underpin the co-design approach used to determine the outcomes, efficacy and structure of the service evaluation. A forthcoming paper will attend to the survey tool itself.

The co-design process described in this paper forms part of an ongoing program of research centred on relationship building between service leaders and Aboriginal Elders. This study is unique in that it demonstrates the leadership of Elders to drive organisational change through the power of relationships and their cultural authority. Many of the co-design workshop participants have a prior history of working together to co-design initiatives and outcomes. One of the limitations of the study is the natural attrition of staff from the study due to organisational restructures, recruitment and succession. This can impact on the continuity of Elder-service relationships and consequently the progress of change made within the organisations. The impact of deeper, long term engagement between service leaders and the Elders has meant that lived experiences and community needs are better understood and solutions are more considered and nuanced to meet these needs. Finally, the formation of relationships occurs at a local level. It is the relationships that develop through this journey of change that give the evaluation its true value. The co-designed service evaluation, we believe, has great potential for use in other settings that aim to assess and enhance the cultural safety of the client’s service experience.

## 6. Conclusions 

The co-design process described above demonstrates how forging and sustaining strong relationships can support service providers, Aboriginal representatives and research stakeholders to come together to develop evaluation measures and data collection instruments that will be more effective in uptake and findings. The underpinning theme of relationships—and the reciprocity that is characteristic of those that are sustained—positions this evaluation as commendable in reflecting broader themes in Aboriginal research and evaluation [67]. Through the administration of the service evaluation, we hope to improve the service experiences of Aboriginal families and create a new paradigm in mental health service provision that celebrates the diversity and contributions of Aboriginal cultures in Australia and globally.

## Figures and Tables

**Table 1:** Strategies and actions reviewed as “draft evidence statements” grouped under the themes of governance, workforce and cultural security, devised during the earlier 2018 working groups.

**Organisational Strategies and Actions, Reviewed as Draft Evidence Statements**	**Governance**	**Workforce**	**Cultural Security**
	quality of the relationship between executive staff and Elders; ways the relationships are prioritised and resourced; Elder and community engagement that includes practical steps in co-design, co-creation and co-production; impact of the ‘storying’ with Elders; more effective communication strategies, use of plain language, and be co-designed with community members; what resources were allocated to share power and influence with Elders and the community; capacity building and ongoing development of staff in understanding culture and history; and ways organisations do and/or could partner with Aboriginal controlled organisations	ways that acknowledge Aboriginal staff cultural and community obligations and skills; organisations required an Aboriginal workforce strategy; targets that the organisation sets in terms of staff numbers (%), positions, roles and responsibilities; service documentation of workforce development efforts; and auditing of recruitment approaches is used to identify and alleviate ‘structural bias and racism’	importance of Elders in setting a foundation for a culturally safe organisation; visual signs of cultural safety in an organisation; client experiences that reflected the relationship-based nature of the work services have undertaken with the Elders; supports for Aboriginal staff (e.g., see also Workforce targets); and supports to create safe spaces and services for clients

## Data Availability

Not applicable.
